# Single stab injuries to the trunk in survivors of corroborated assaults

**DOI:** 10.1007/s00414-025-03629-5

**Published:** 2025-10-23

**Authors:** Maria Berg von Linde, Stefan Acosta, Ardavan M. Khoshnood, Carl Johan Wingren

**Affiliations:** 1 Present Address: Unit for Forensic Medicine, Department of Clinical Sciences Malmö, Faculty of Medicine, Lund University, Malmö, Sweden; 2 Unit for Forensic Medicine, Swedish National Board of Forensic Medicine, Sölvegatan 25, Lund, 223 62 Sweden; 3 Vascular Centre, Department of Cardiothoracic and Vascular Surgery, Skåne University Hospital, Malmö, Sweden; 4 Department of Clinical Sciences Malmö, Lund University, Ruth Lundskogsgatan 10, Malmö, 205 02 Sweden; 5 Department of Emergency Medicine, Skåne University Hospital Malmö, Malmö, Sweden; 6 Department of Clinical Sciences Malmö, Clinical Research Centre, Lund University, CRC 91-12, Box 50332, Malmö, 202 13 Sweden; 7 Department of Forensic Medicine, University of Copenhagen, Frederik V’s Vej 11, Copenhagen, 2100 Denmark

**Keywords:** Stab, Sharp force, Assault, Forensic medicine

## Abstract

**Introduction:**

The evidence for assessing whether a single stab injury to the trunk was inflicted by another person in an assault or self-inflicted has primarily been derived from autopsy studies. In this study, we investigate whether victim demographics, crime scene circumstances, and the injury characteristics of assault survivals are interchangeable with homicide characteristics, with a specific focus on cases corroborated by perpetrator confession or eyewitnesses.

**Methods and materials:**

Surviving victims of assaults including single stab injuries to the trunk were identified in a registry including all cases of clinical forensic medicine assessed by the Swedish National Board of Forensic Medicine between the years 2016 and 2021. Characteristics were compared between corroborated and non-corroborated survivors of assault. We also used a reference population of homicide victims with single stab wounds to the trunk.

**Results:**

Survivors of corroborated (*n* = 162) and non-corroborated assaults (*n* = 223) showed largely similar victim demographics and injury characteristics. Compared to fatal cases, survivors more frequently experienced stabs to the abdomen, left axillary region, and the back, and, in fewer cases, to the bony part of the ribcage.

**Discussion and conclusion:**

Similar findings among corroborated and non-corroborated assault survivors suggest that most non-corroborated cases likely involve victims of actual assaults. According to our results, injury characteristics do not seem to be directly interchangeable between fatal and survived assaults, due to the differences in injury severity, which highlights the need for cautious interpretation of autopsy-based characteristics in living victims.

**Supplementary Information:**

The online version contains supplementary material available at 10.1007/s00414-025-03629-5.

## Introduction

 Evidence-based characteristics applied to assess whether a single stab wound is self-inflicted, accidental, or inflicted by another person are essentially based on findings from autopsy populations [1–4]. Hence, the evidence base stems from populations with fatal stab wounds, which is accordingly potentially flawed by selection bias. The findings may, therefore, not be directly applicable to victims who have survived stab wounds. Moreover, in such autopsy-based populations, the manner of death—suicide, accident or homicide—is based on the assessment made by the forensic pathologist, possibly also introducing a circularity bias.

Studies on sharp force injuries comparing assaults and self-inflicted injuries among surviving victims typically originate from trauma settings [5–10], where the primary focus is on treatment and prevention. Only a few studies of survivors of sharp force injuries involve forensic aspects such as injury patterns [11] and crime scene locations [8, 9]. Findings in these studies show some parallels with findings in autopsy populations [1–4]; a predominance of male victims [8, 9, 11], and lower mean age among cases of assault compared to cases involving self-inflicted injuries [8, 9]. Furthermore, victims of assault were more frequently suffering from alcohol inebriation [9], and assaults often occurred in a public area [8, 9]. Survivors of assaults had defensive injuries [11], and a notable proportion had injuries to the back [11]. However, several essential variables remain unexplored in survivals of assault, including where the weapon is found, the location and orientation of the entrance wound, and whether the stab wound passes through the bony parts of the ribcage or the intercostal space. Another issue with studying populations obtained in a healthcare setting may be the increased risk of misclassification of mode of origin when there is no access to police investigation or a forensic assessment. Victim groups presenting a challenge to forensic assessment are cases involving self-mutilated sharp force injuries reported to the police as an assault [12], and cases with stab wounds that refuse to cooperate with the police on the origin of the injuries.

In other areas in forensic medicine, such as in abusive head trauma in infants, there have been efforts to expand evidence-based forensic medicine beyond relying on conclusions based only on expert opinions as the gold standard [13, 14]. To further close the knowledge gap in evidence-based forensic medicine, suggestions have been made to increase the certainty on how injuries are inflicted, by accounting for filmed, witnessed, or confessed cases.

No study to date has specifically investigated single stab injuries in survivors of assault. In this study, we aim to investigate surviving cases of single stab wounds to the trunk by analysing findings previously known to be associated with fatal single stabs to the trunk. Moreover, by focusing specifically on cases both classified as assaults by forensic pathologists and supported by corroborating evidence, such as eyewitness or confession by the perpetrator, we aim to strengthen the evidence base for forensic assessment.

## Materials and methods

### Study population

We identified all victims exposed to a single stab injury to the trunk in the registry of clinical forensic assessments kept by the Swedish National Board of Forensic Medicine between the years 2016 and 2021. Victims included in this registry are police-reported cases in which the police wanted a complete documentation of injuries alongside a forensic report. Swedish forensic reports include statements issued by the forensic pathologists based on the forensic body examination, and medical records from the healthcare visit following the stabbing incident and/or photographic documentation of the injuries, if present. In many instances, the forensic reports do not include a forensic body examination, relying instead on the clinical medical record and/or photographic documentation.

We searched the forensic reports for the Swedish terms representing the keywords “stab wound”, “knife stab”, “sharp edged”, “pleura”, “stab channel”, “stabbing”, “egg sharp”, “knife-like”, “knife slash”, and “knife cut”. In total, 9162 reports (cases) were identified. The first author (MBvL) manually reviewed these cases and included all victims aged at least 15 years with a single stab injury to the trunk. Sharp injuries caused by a combination of incision and stab wounds and/or with more than one direction of the stab wound channel, were still considered a single stab wound and were included in the study. Furthermore, we included stab injuries with entrance wounds placed below the clavicula and above the os pubis. Cases with more than one stab injury to the body were excluded. Cases with incisive wounds, regardless of depth, and blunt force injuries beyond the stab injury were still included.

As a reference we included homicide cases caused by single stabs to the trunk between the years 2010 to 2021. The homicide cases were previously identified in the autopsy register kept by the Swedish National Board of Forensic Medicine. The process of including the study population and the data collection from these cases is explained in detail in a previous article for this population [4].

### Collection of variables from clinical forensic cases

Information on demographics, crime scene, injuries and the forensic conclusion was extracted from documents accessible in the clinical forensic medicine registry maintained by the Swedish National Board of Forensic Medicine, including police reports, police interrogations, medical records, image material and forensic reports.

The causation of the stab injuries was defined in accordance with the assessment in the forensic report issued by the forensic pathologist as either: (i) assault, (ii) self-inflicted, (iii) accidental, or (iv) not assessable.

Based on the information in police reports, it was noted whether a suspected perpetrator (i) had confessed (ii) or not, and if the suspected crime was (i) witnessed (ii) or not. If the suspected perpetrator claimed self-defence or that he/she had accidentally stabbed the plaintiff during a tumultuous fight, the testimony was still categorised as a confession. Cases with no information about confessions or available witnesses in the material sent to the forensic pathologists were categorised as not confessed and not witnessed. Furthermore, cases in which the stabbings were either confessed or witnessed were defined as corroborated assaults and cases in which the stabbing were neither confessed nor witnessed were categorised as non-corroborated assaults.

Age in years was included as a continuous variable and gender was defined from their designation in the police reports.

Psychiatric diagnosis and abuse of alcohol and/or narcotics were categorised as (i) present when mentioned in the medical records supplied for the forensic assessment, or (ii) absent when medical records were available but made no mention of them. Otherwise, it was classified as missing.

The crime scene was categorised into: (i) the victim’s home, (ii) an inside location other than victim’s home, or (iii) an outside location. The garden or courtyard outside the victim’s home was defined as an outside location and, e.g., the stairwell outside the apartment was defined as an inside location other than the victim’s home.

Information about the weapon which might have been used to cause the stab injury was categorised into (i) object found in situ in the body at the time of arrival of the police or ambulance, (ii) object recovered from the crime scene, (iii) recovered from the suspect, or (iv) no information about a recovered object.

Information about the clothing on the trunk was categorised into: (i) injury through clothing, (ii) no injury through clothing, or (iii) no clothing on the trunk. Cases without information about the victim’s clothing on the upper body and cases in which the clothing had not been examined were categorised as missing.

We documented the influence of alcohol and narcotics as the victims arrived at the hospital after the stabbing incident. The results were categorised into: (i) positive or (ii) negative alcohol results; and (i) positive or (ii) negative illicit narcotics, licit narcotics, or unspecified narcotic results. Narcotics considered illicit were tetrahydrocannabinol, amphetamine, cocaine, and heroin. The influence of ethanol or narcotics was considered positive when the medical records showed a positive sample of such a substance or when the victims stated that he/she was under the influence of alcohol or narcotics during the stabbing incident.

The anatomical placement of the entrance wound was partitioned into: (i) anterior trunk, (ii) right axillary region, (iii) left axillary region, or (iv) posterior side of the trunk. The anterior and posterior trunk was also divided into: (i) right side, (ii) left side, and (iii) vertical midline (including injuries placed in line with or in proximity to the midline, take injury to the sternum as an example). The more precise positions of the injuries were orientated according to midline, midclavicular, and axillary lines, skeletal structures, and umbilicus.

The stab injuries were categorised into: stabs (i) perforating the thoracic wall, (ii) the abdominal wall, or (iii) superficial stabs not perforating into the thoracic or abdominal cavity. We also documented whether stab injuries penetrating the thoracic wall involved: (i) the skeleton or cartilage of the ribcage, such as the ribs(s)/sternum/scapula, or (ii) whether they penetrated the intercostal space.

The orientation of the stab injury’s entrance wound in the skin was categorised into: (i) horizontally, (ii) vertically, (iii) diagonally downward-right, or (iv) diagonally downward-left orientated.

The depth of the injury in cm was included as a continuous variable.

The orientation of the injury channel was classified based on the sagittal and horizontal plane. In the sagittal plane, the channel was categorised as: (i) cranially, (ii) caudally, or (iii) straight horizontally orientated; and in the horizontal plane, orientation was classified as either (i) medially, (ii) laterally, or (iii) straight sagittally orientated.

Superficial sharp injuries were classified as defensive or hesitation injuries if stated as such injuries in the forensic report or if there were sharp injuries of typical appearance/description and location. Typical defensive injuries were identified by fresh sharp injuries to the palms, or the ulnar sides of the forearms, and typical hesitation injuries were characterised by fresh parallel superficial incisive injuries on the extensor sides of the forearms. The defensive and hesitation injuries were partitioned into: (i) present or (ii) absent.

## Statistics

Categorical variables were presented in numbers and percentages, and the differences between corroborated and non-corroborated assaults were analysed using the Fisher’s exact test. Continuous variables were presented using median and range, and comparison between corroborated and non-corroborated assaults were tested using Mann-Whitney U test. *P* < 0.05 was considered statistically significant.

**Fig. 1:**
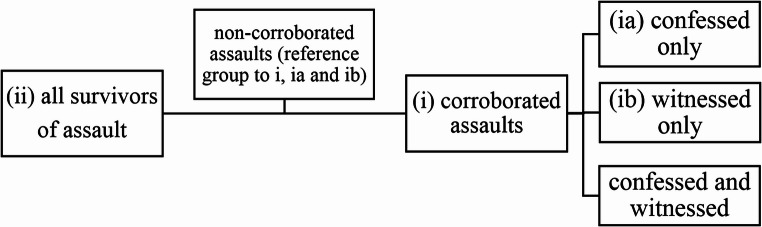
Grouping of assault survivors for analyses

We performed univariate logistic regression analysis for evaluation of differences in demographics, circumstances, and injury characteristics between: (i) survivors of corroborated and non-corroborated assaults, and (ii) survival of assaults and autopsied homicide population (Fig. 1). We conducted subgroup analyses, examining differences in the specified variables between (i.a.) survivors of confessed (excluding witnessed assaults) and non-corroborated assaults, as well as between (ib) survivors of witnessed (excluding confessed assaults) and non-corroborated assaults.

A directed acyclic graph (Supplementary Figure S1) was applied to identify an appropriate multivariable model that adjusts for potential confounding in the multivariable analysis [15]. The covariates included in the multivariable regression analyses, examining differences between (i) corroborated and non-corroborated survived assaults, included age, sex, psychiatric diagnosis, and alcohol and/or narcotic abuse. Psychiatric diagnoses and alcohol and/or narcotic abuse were not included as covariates in the multivariable logistic regression model comparing (ii) survived assaults with homicides, due to a high proportion of missing data for these variables among homicide cases (83% and 73%, respectively) [4].

The data analyses were performed using IBM SPSS Statistics Premium 28. Odds ratios (OR) were expressed with 95% confidence intervals (CI).

## Results

### Study population of corroborated and non-corroborated assaulted survivors

**Fig. 2:**
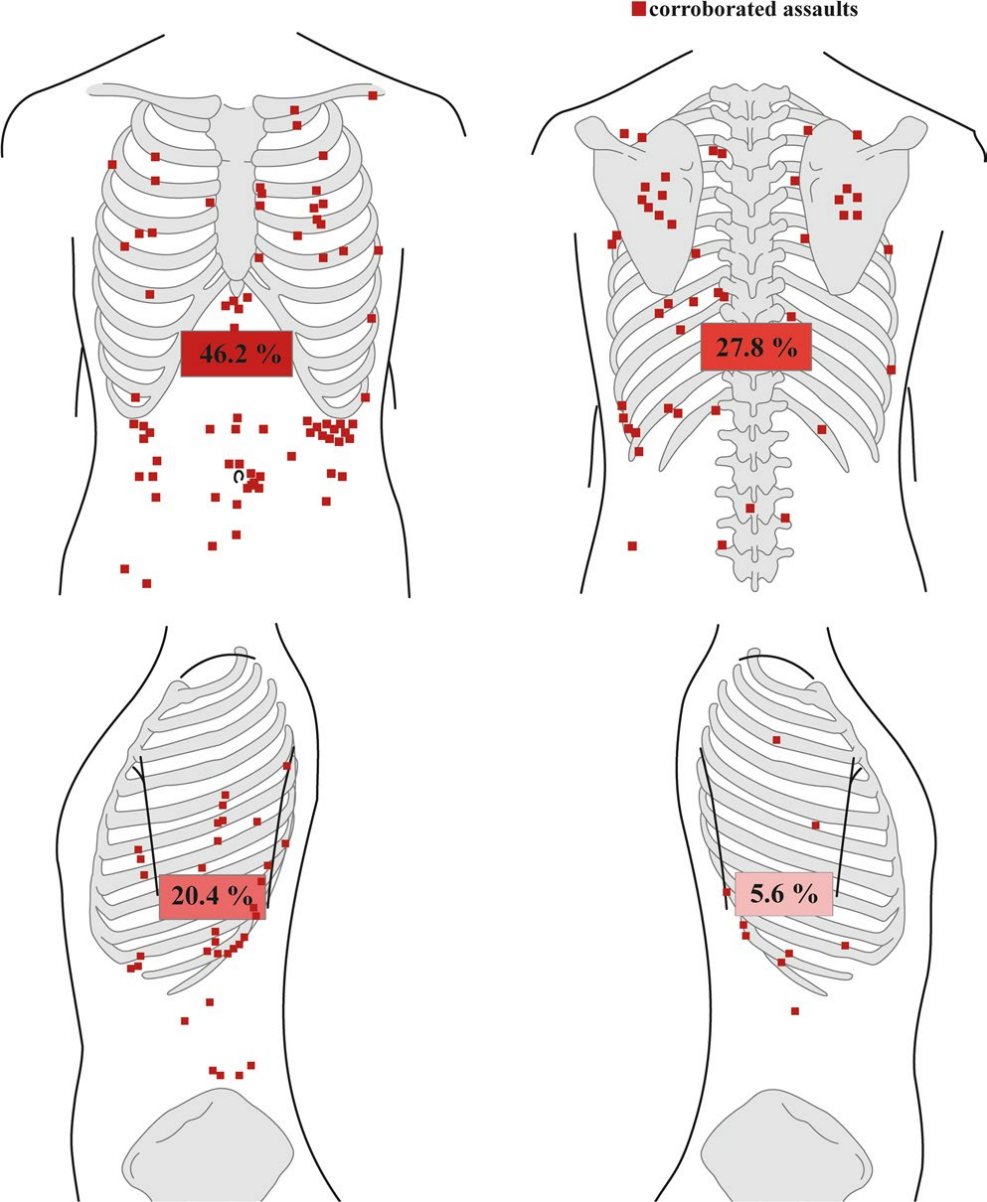
Anatomical location of the entrance wounds in survivors of corroborated assaults

The study population consisted of 385 cases that survived a stab wound to the trunk inflicted during an assault. These cases were divided into groups of corroborated assaults (*n* = 162) and non-corroborated assaults (*n* = 223). We analysed the population for demographics (Table 1); circumstances surrounding the assault (Table 2); and injury characteristics, including anatomical location (Table 3; Fig. 2). A total of 23 cases with self-inflicted, accidental, and non-assessable stabs were excluded from the analyses.

**Table 1:** Demographics and background of the study population

	Corroborated assaults	Non-corroborated assaults	*P*-value
Number of cases, n (%)	162 (100)	223 (100)	
Males, n (%)	151 (93.2)	210 (94.2)	
Females, n (%)	11 (6.8)	13 (5.8)	*p* = 0.8
Age in years, median (range)*	32 (15–79)	29 (15–76)	*p* = 0.07
	*n* = 161		
Psychiatric diagnosis, n (%)*	22/161 (13.7)	24/218 (11.0)	*p* = 0.4
Alcohol abuse, n (%)*	12/161 (7.5)	22/218 (10.1)	*p* = 0.5
Narcotic abuse, n (%)*	31/161 (19.3)	43/218 (19.7)	*p* = 1.0

Gender and presence of psychiatric diagnosis and abuse were presented in numbers and percentages and the differences between the corroborated and non-corroborated assaults were estimated using Fisher´s exact test. Age was presented as median and range in years and difference between the two groups was estimated using Mann-Whitney U test

*Missing values: age n = 1 (0.3%), psychiatric diagnosis, alcohol and narcotic abuse n = 6 (1.6%)

**Table 2:** Crime scene circumstances and toxicological findings of the victims

Variables, n (%)	Corroborated assaults	Non-corroborated assaults	*P*-value
Injury inflicted at home*	39/161 (24.2)	41/201 (20.4)	*p* = 0.4
Other indoor location*	48/161 (29.8)	39/201 (19.4)	*p* = 0.03
Outdoors*	74/161 (46.0)	121/201 (60.2)	*p* = 0.008
Object found in situ	≤ 3 (1.2)	6 (2.7)	*p* = 0.5
Object found at the scene	53 (32.7)	43 (19.3)	*p* = 0.003
Object found within possession of the suspect	22 (13.6)	11 (4.9)	*p* = 0.005
No object found	85 (52.5)	163 (73.1)	*p* < 0.001
Injury in clothing	26/27 (96.3)	23/25 (92.0)	
No injury in clothing	≤ 3/27 (3.7)	≤ 3/25 (8.0)	*p* = 0.6
No clothing	0/27 (0)	0 (0)	
Influence of alcohol	52 (32.1)	47 (21.3)	*p* = 0.02
Illicit narcotics	9 (5.6)	14 (6.3)	*p* = 0.8
Licit narcotics	≤ 3 (1.2)	7 (3.2)	*p* = 0.3
Unspecified narcotics	≤ 3 (0.6)	5 (2.3)	*p* = 0.4

Variables involving crime scene location, weapon, clothing and findings of alcohol and narcotics were presented in numbers and percentages. The differences between corroborated and non-corroborated assaults were estimated using Fisher´s exact test

*Missing values: crime scene location n = 23 (6.0%), information on clothes n= 333 (86.5%)

**Table 3:** Injury characteristics of the victims

Variables, n (%)	Corroborated assaults	Non-corroborated assaults	*P*-value
Penetrating thoracic wall	75 (46.3)	93 (41.7)	*p* = 0.4
Penetrating abdominal wall	53 (32.7)	50 (22.4)	*p* = 0.03
Non-penetrating	34 (21.0)	80 (35.9)	*p* = 0.002
Penetrating the bones of the ribcage*	22/55 (40.0)	24/65 (36.9)	
Penetrating the intercostal space*	33/55 (60.0)	41/65 (63.1)	*p* = 0.4
Vertical entrance wound*	27/86 (31.4)	27/128 (21.1)	*p* = 0.1
Horizontal entrance wound*	23/86 (26.7)	45/128 (35.2)	*p* = 0.2
Downward right*	15/86 (17.4)	28/128 (21.9)	*p* = 0.5
Downward left*	21/86 (24.4)	28/128 (21.9)	*p* = 0.7
Length of the injury channel, median (range) (cm)*	5.5 (0.3–17.5)	4.5 (0.6–23.0)	*p* = 0.05
	*n* = 33	*n* = 43	
Cranial injury channel*	12/37 (32.4)	11/45 (24.4)	*p* = 0.5
Caudal injury channel*	25/37 (67.6)	33/45 (73.3)	*p* = 0.6
Straight horizontal injury channel*	0 (0)	≤ 3/45 (2.2)	*p* = 1.0
Medial injury channel*	36/42 (85.7)	37/43 (86.0)	*p* = 1.0
Lateral injury channel*	5/42 (11.9)	6/43 (14.0)	*p* = 1.0
Straight sagittal injury channel*	≤ 3/42 (2.4)	0 (0)	*p* = 0.5
Defensive injuries	7 (4.3)	21 (9.4)	*p* = 0.07
Hesitation injuries	0 (0)	≤ 3 (0.4)	*p* = 1.0

Variables involving injury penetrating the thoracic or abdominal cavities, injury to the ribcage, orientation of the entrance wound, orientation of the injury channel in the sagittal and horizontal plane, presence of defensive and hesitation injuries were presented in numbers and percentages, and the differences between corroborated and non-corroborated were estimated using Fisher´s exact test. Length of the injury channel was presented as median length (cm) and range and difference between corroborated and non-corroborated assaults was estimated using a Mann-Whitney U test

*Missing values: injury to the ribcage n=265 (68.8%), direction of the entrance wound n=171 (44.4%), length of the injury channel n=309 (80.3%), direction of the injury channel, sagittal plane n=303 (78.7%) and horizontal plane, n=300 (77.9%)

.

The majority of victims were males, with 93.2% of corroborated assaults and 94.2% of non-corroborated assaults (Table 1). The median age for corroborated cases was 32 years and 29 years for non-corroborated assaults.

Psychiatric diagnoses were identified in 13.7%, alcohol abuse in 7.5%, and narcotic abuse in 19.3% of corroborated assaulted cases, with no significant differences compared to the non-corroborated assaulted group (Table 1).

### Circumstances at the crime scene

The crime scenes were predominantly located outdoors. The proportion of cases with an outdoor crime scene was significantly less prevalent in corroborated cases (46.0%) compared to non-corroborated cases (60.2%) (Table 2). A significantly higher number of corroborated cases (29.8%) were stabbed at an indoor location other than their homes compared to non-corroborated assaults (19.4%). In corroborated assaults, the weapon was significantly more often found at the crime scene (32.7% vs. 19.3%) or in the suspect’s possession (13.6% vs. 4.9%) than in non-corroborated assaults. In non-corroborated assaults, information on the object used was more often lacking (73.1% vs. 52.5%). Among cases with information on clothing in connection with the stab wounds (*n* = 52), nearly all cases of assault had a cut or slash in the fabric of the clothing. In the few cases (*n* ≤ 3) without corresponding clothing damage, we could not identify any data in the reports on how this finding was handled in the case assessment.

Of the corroborated cases, 32.1% were positive for alcohol when arriving at the hospital, significantly higher than the 21.3% of non-corroborated cases. Illicit narcotics was positive in 5.6% of corroborated and 6.3% of non-corroborated assaults.

### Anatomical location

The specific anatomical locations of the entrance wounds in corroborated assaults are illustrated in Fig. 2. Almost half of the entrance wounds in corroborated assaults (46.2%) were anatomically located in the frontal trunk, with 17.9% in the frontal thorax and 28.4% in the abdomen. Also, 27.8% of entrance wounds were located in the back, 5.6% in the right axillary region and 20.4% in the left axillary region. The non-corroborated assaulted cases (not shown in the figure) showed similar proportions of stab wounds located to the frontal trunk, back, and right axillary region but significantly (*p* = 0.02) fewer stab wounds to the left axillary region (11.7%).

### Injury characteristics

There were significantly more stab wounds penetrating the thoracic- or abdominal cavity among corroborated victims (*p* = 0.002) compared to non-corroborated victims, with a similar proportion penetrating the thoracic cavity (46.3% vs. 41.7%), whereas there were significantly more stab wounds penetrating the abdominal cavity among the corroborated victims (32.7% vs. 22.4%). In victims with injuries to the thoracic wall, the majority of stab wounds penetrated the intercostal space in both groups (60.0 vs. 63.1%).

Stab wounds in corroborated cases were significantly deeper compared to non-corroborated cases, with medians of 5.5 cm and 4.5 cm, respectively. In the sagittal plane, the majority of both groups had caudally orientated injury channels (67.6% vs. 73.3%), and in the horizontal plane, the majority of injury channels were of a medial direction in both groups (85.7% vs. 86.0%).

Defensive wounds were reported in 4.3% of the corroborated assaults and in 9.4% of the non-corroborated assaults with no significant difference (*p* = 0.07) between the groups.

### Variables associated with all corroborated, confessed, and witnessed assaults, in relation to non-corroborated assaulted survivors

In the univariable logistic regression model analysing retrieved weapons, cases in which an object was retrieved at the crime scene or was in the possession of the suspected perpetrator showed conclusive associations with corroborated cases of survived assaults (OR 2.4, 95% CI 1.5–3.8, and OR 3.8, 95% CI 1.8–8.3, respectively) and with confessed assaults alone (OR 4.4, 95% CI 2.3–8.2, and OR 4.0, 95% CI 1.4–11.2, respectively) (Table 4). Objects found in the subject’s possession were conclusively associated with witnessed assaults as opposed to non-corroborated cases (OR 3.5, 95% CI 1.5–8.4).

**Table 4:** Variables conclusively associated with all corroborated, confessed, or witnessed assaults

	All corroborated assaults, *n* = 162 (ref non-corroborated assaults, *n* = 223), OR (95% CI)		Subgroup analyses (ref non-corroborated assaults, *n* = 223), OR (95% CI)			
	**Univariable model**	**Multivariable model**	**Confessed assaults alone**, *n* = 64	**Witnessed assaults alone**, *n* = 82		
Other indoor location	1.3 (0.7–2.4)	1.3 (0.7–2.5)	0.8 (0.4–1.7)	2.9 (1.2–7.0)		
Outdoors	0.6 (0.4–1.1)	0.7 (0.4–1.3	0.3 (0.1–0.5)	1.8 (0.8–4.0)		
Injury inflicted at home	Ref	Ref	Ref	Ref		
Object found in situ	0.6 (0.1–3.2)	0.5 (0.1–2.9)	1.0 (0.1–9.0)	0.5 (0.1–4.5)		
Object found at the scene	2.4 (1.5–3.8)	2.2 (1.4–3.7)	4.4 (2.3–8.2)	1.3 (0.7–2.5)		
Object found in suspect’s possession	3.8 (1.8–8.3)	3.7 (1.7–8.1)	4.0 (1.4–11.2)	3.5 (1.5–8.4)		
No object found	Ref	Ref	Ref	Ref		
Alcohol influence	1.8 (1.1–2.8)	1.8 (1.1–2.9)	2.1 (1.1–3.8)	1.3 (0.7–2.3)		
No alcohol influence	Ref	Ref	Ref	Ref		
The back	1.2 (0.7–1.9)	1.2 (0.7–1.9)	1.7 (0.9–3.2)	0.9 (0.5–1.8)		
Right axillary region	1.1 (0.5–2.7)	1.1 (0.4–2.6)	0.7 (0.2–3.5)	1.7 (0.6–4.5)		
Left axillary region	2.0 (1.1–3.7)	2.1 (1.1–3.8)	2.8 (1.3–6.0)	2.0 (1.0–4.1)		
Frontal trunk	Ref	Ref	Ref	Ref		
Penetrating thoracic wall	2.0 (1.2–3.4)	1.9 (1.1–3.2)	1.7 (0.8–3.6)	2.3 (1.2–4.6)		
Penetrating abdominal wall	2.5 (1.4–4.4)	2.5 (1.4–4.5)	2.2 (1.1–4.7)	2.5 (1.2–5.1)		
Non-penetrating	Ref	Ref	Ref	Ref		
Vertical entrance wound	2.0 (0.9–4.1)	1.7 (0.8–3.7)	2.7 (1.1–6.7)	2.0 (0.7–5.5)		
Down–right-oriented entrance wound	1.0 (0.5–2.3)	0.9 (0.4–2.1)	1.3 (0.5–3.6)	1.1 (0.3–3.3)		
Down–left-oriented entrance wound	1.5 (0.7–3.1)	1.3 (0.6–2.9)	1.6 (0.6–4.3)	1.8 (0.6–4.9)		
Horizontal entrance wound	Ref	Ref	Ref	Ref		

Univariable logistic regression models presenting associations between variables and corroborated assaults, confessed assaults alone and witnessed assaults alone using non-corroborated assaults as a reference and a multivariable logistic regression model, adjusting for gender, age, psychiatric diagnosis, and alcohol/narcotic abuse, demonstrating associations between variables and corroborated assaults (ref non-corroborated assaults). Odds ratios (OR) are presented with 95% confidence intervals (CI)

When analysing crime scene location separately for witnessed assaults, indoor locations other than the victim´s home (OR 2.9, 95% CI 1.2–7.0) were conclusively associated with witnessed assaults. Outdoor crime scene locations were negatively associated with confessed assaults (OR 0.3, 95% CI 0.1–0.5).

Being under the influence of alcohol at the time of the stabbing was conclusively associated with corroborated assaults and with confessed assaults alone (OR 1.8, 95% CI 1.1–2.8, and OR 2.1, 95% CI 1.1–3.8, respectively).

Stab wounds in the left axillary region (OR 2.0, 95% CI 1.1–3.7) and ones penetrating the thoracic or abdominal cavity (OR 2.0, 95% CI 1.2–3.4, and OR 2.5, 95% CI 1.4–4.4) were conclusively associated with corroborated assaults.

The multivariable model demonstrated point estimates and confidence intervals closely mirroring those of the univariate model (Table 4 and S1, respectively).

Variables with no significant differences between all corroborated assaults, confessed assaults, and witnessed assaults—as opposed to non-corroborated assaults—are presented in Table S1.

### Variables associated with survived assaults in relation to homicide victims

In the univariable logistic regression model comparing survived assaults to homicides, victims with psychiatric diagnoses, a history of alcohol and narcotic abuse, or who were under the influence of alcohol and narcotics, as well as penetration of the bony part of the ribcage, and a downward and to right orientation of the entrance wound, showed conclusive negative associations with survived assaults, using homicides as the reference (Table S2).

Injuries located in the abdomen rather than in the thorax and to the back or the left axillary region showed conclusive associations with survived assaults compared to homicides (Table S2).

The results of the multivariable model showed point estimates and confidence intervals similar to those from the univariate analyses (Table S2 and S3, respectively).

Variables with no significant difference between survived assaults and homicides are presented in Table S3.

## Discussion

This study presents, to our knowledge, the first forensic investigation on corroborated single stab injuries to the trunk in survived assault victims. By focusing on cases with corroborative evidence, we aimed to reduce the limitations of circulatory bias that have characterised previous studies which are primarily based on homicide populations [1–4, 8, 9, 11].

The survivors of assaults of single stab wounds to the trunk comprised a young, male dominated population, with similar demographics as those seen in previous studies on populations with homicidal single stabs [1–4]. Also, most of the assaulted victims were stabbed outdoors with no weapon to be found, as described previously [4, 8, 9]. Victims with psychiatric diagnoses, and a history of alcohol and narcotic abuse were more prevalent in the reference group of homicides [4] compared to those who survived their assaults. However, a substantial number of medical records involving past medical history were missing in the homicide cases, since victims were found deceased at the scene or died shortly after hospital arrival [4]. It is possible that records with previous medical history were requested to a greater extent in cases involving a prior suspicion of psychiatric illness or abuse, potentially leading to a disproportionately high percentage of positive findings in homicide victims. In contrast, medical history is typically documented in the records of assault survivors receiving hospital treatment, as it may be considered relevant to their clinical care and future follow-up.

Also, homicide victims with single stab wounds were more frequently inebriated by either alcohol (48–58%) or illicit narcotic substances (14–36%) [3, 4] during the assault compared to the survivors in the present study (32% influenced by alcohol and 6% by narcotics). The lower proportion of inebriated assaulted survivals could, however, be an underestimation, as testing for ethanol and narcotics does not seem to have been routinely performed at emergency departments [16], in contrast to routine sampling during autopsy.

Previous studies have reported that defensive injuries occur in 3%–33% of victims assaulted with a single stab wound [1, 2, 4, 11, 17, 18]. Our population of surviving assaults were at the lower end of this range (4% in corroborated and 9% in non-corroborated assaults). However, a significant number of victims in our population did not undergo a forensic examination and were only evaluated based on hospital charts. As a result, cases with defensive injuries may have been overlooked or simply not documented during the hospital visit, underscoring the importance of forensic examinations.

Some differences were observed when comparing the anatomical location of single stabs in assaulted survivors with homicides. As expected, abdominal stab wounds, as opposed to thoracic stab wounds, were associated with survived assaults compared to homicides. The greater distribution of stabs to the abdomen in survivors (28%) was in line with findings in previous studies on homicides (10%–18% abdominal stabs) [1, 3, 4, 19]. This could be explained by the generally lower severity of damage when abdominal organs are injured, compared to stab wounds to the heart or to great vessels in the thorax [20]. Among the single stabs penetrating the thoracic wall, fewer injuries penetrating the bony parts of the ribcage were seen in assaults (40%) compared to homicides (74%–87%) [1, 4]. This could be due to the fact that stab wounds with enough force to penetrate bone are more likely to cause severe injuries to the thoracic organs and involve a fatal outcome. The bones in the ribcage could possibly protect against stab wounds of lesser force, thereby categorising the stab wounds as non-penetrating. Previous studies on homicide populations have found that only 0%–13% of stab wounds were located in the back [1–4, 19], which can be compared to 28% in our population of survivors. These findings strongly imply that the skeletal structures of the ribcage and scapulae prevent penetration of objects to vital organs.

We found similar victim demographics and injury characteristics among corroborated and non-corroborated assaults, other than more stab wounds located to the left axillary region and those penetrating the thoracic and abdominal cavities in corroborated cases. Most importantly, variables typical of self-inflicted injuries, such as victims with psychiatric diagnoses [5, 6, 9, 10], weapons still in situ of the body [3, 4], injuries without corresponding damage to clothing [1–4], stab wounds to the anterior trunk [1–4], and the presence of hesitation wounds [1, 2, 4], did not show significant differences between corroborated and non-corroborated assaults. The few cases of corroborated assaults in which intact clothing was observed in association with the stab lesion, underscore that absence of clothing damage alone is not sufficient to classify an injury as self-inflicted. Thus, the possibility of a significant share of non-corroborated assaults actually being cases of self-mutilated injuries falsely reported as assault is not supported by our findings. Instead, the results indicate that both corroborated and non-corroborated cases likely include genuinely assaulted victims.

However, some circumstantial differences were seen between the subgroups of assaulted survivors. Assaults in which the suspect confessed were associated with inebriated victims, attacks occurring in the victims’ homes, and with the weapon being found either at the scene or in the suspect’s possession. This pattern may suggest that acts of domestic violence under the influence of alcohol are more likely to result in confession. Furthermore, early retrieval of strong evidence, such as a weapon capable of yielding fingerprints or DNA, has been shown to increase the likelihood of a confession [21]. Witnessed assaults more commonly occurred in indoor locations other than the victim’s home with the weapon found in the possession of the suspect, suggesting that such settings increase the likelihood of observation by others, which in turn facilitates suspect identification, arrest, and confiscation of weapons from the suspect.

## Strengths and limitations

A main strength of this study lies in focusing on cases classified as assaults by the conclusion of forensic pathologists together with additional corroborating evidence. This approach helps reduce the risk of circular reasoning, where case classification is determined solely by the forensic pathologist. In such instances, conclusions may be influenced by prior interpretations and lack independent, objective verification.

By highlighting cases with additional evidence, such as confessed or witnessed assaults, our findings provide improved reliability for identifying injury characteristics associated with assault rather than self-mutilated injuries. Nevertheless, there are also limitations to this approach. First, confessions or witnesses are not equivalent to complete security regarding the event, as these accounts could be fabricated or incomplete. Secondly, in our study we only had access to confessions and witnesses documented at an early stage of the police investigation, which suggests that there could be testimonies claiming a different event or withdrawals of confessions or witnesses at a later stage of the investigations that were never documented in the registry used for this study.

There are also methodological limitations which merit consideration. Unfortunately, we did not manage to collect a proper reference group for the assaults, which would optimally be a population of survived self-inflicted stab wounds. The forensic registry contained too few cases within the specified time interval to serve as a reliable reference group. Another weakness is the retrospective approach, where there was a variability of documentation between cases in the forensic registry. Information about stab wounds through clothing, the orientation of the entrance wound, and the depth and direction of the wound channel were missing in a large number of cases. Additionally, information on alcohol and narcotic use could only be obtained from medical records, in contrast to prospective studies [22], or at institutions where blood samples are routinely collected during physical forensic examinations of survivors.

## Conclusion

This study provides evidence to support the forensic evaluation of single stab injuries in victims surviving assault, highlighting corroborated cases to reduce classification bias. The findings in surviving stab victims overlap only partially with those seen in homicides, but with more stab wounds to the abdomen, back, and left axillary region and fewer stab wounds penetrating the bony parts of the ribcage in injury survivors. These differences emphasise difficulties in assuming interchangeability between findings in survived and fatal cases. Further research should aim to expand the comparative analysis to also include self-inflicted single stab wounds in survivals, preferably including full forensic examinations of victims.

## Supplementary Information

Below is the link to the electronic supplementary material.

Supplementary Material 1 (DOCX 50.4 KB)

Supplementary Material 2 (DOCX 20.8 KB)

Supplementary Material 3 (DOCX 17.5 KB)

Supplementary Material 4 (DOCX 16.4 KB)
