# Supplementary material for: Single stab injuries to the trunk in survivors of corroborated assaults
Source: Int J Legal Med. 2025 Oct 23;140(2):1121–30. doi: 10.1007/s00414-025-03629-5 (PMC12957011; doi:10.1007/s00414-025-03629-5)
Supplement: Supplementary file 1 — Supplementary Material 1 (DOCX 50.4 KB) [file 414_2025_3629_MOESM1_ESM.docx]

## **Supplemental material**

**Fig. S1 A directed acyclic graph illustrating potential confounders in the analysis of associations between the circumstances at the crime scene and injury characteristics and corroborated assaults (ref non-corroborated assaults) and all survived assaults (ref homicides), respectively**

When adding in the circumstances at the scene and injury characteristics as exposures on the outcomes of corroborated survived assaulted victims (ref non-corroborated survived assaulted victims) and all survived assaulted victims (ref homicide victims) age, sex, psychiatric diagnosis, and alcohol and/or narcotic abuse could be identified as potential confounders.

**Article title:** Single Stab Injuries to the Trunk in Survivors of Corroborated Assaults

**Journal name:** International Journal of Legal Medicine

**Author names:** Maria Berg von Linde, MD, Stefan Acosta, MD, PhD, Ardavan M. Khoshnood MD, PhD, Carl Johan Wingren, MD, PhD.

**Affiliation and e-mail address of the corresponding author:** Maria Berg von Linde, MD, Unit for Forensic Medicine, Department of Clinical Sciences Malmö, Faculty of Medicine, Lund University, Sweden. Electronic address: [maria.berg_von_linde@med.lu.se](mailto:maria.berg_von_linde@med.lu.se)
