# Supplementary material for: Single stab injuries to the trunk in survivors of corroborated assaults
Source: Int J Legal Med. 2025 Oct 23;140(2):1121–30. doi: 10.1007/s00414-025-03629-5 (PMC12957011; doi:10.1007/s00414-025-03629-5)
Supplement: Supplementary file 2 — Supplementary Material 2 (DOCX 20.8 KB) [file 414_2025_3629_MOESM2_ESM.docx]

**Table S1. Variables with no conclusive associations with corroborated, confessed, or witnessed assaulted survivors**

|  | All corroborated assaults, n =162 (ref non-corroborated assaults, n =223),  OR (95% CI) | | Subgroup analyses (ref non-corroborated assaults, n =223),  OR (95% CI) | |
| --- | --- | --- | --- | --- |
|  | **Univariable model** | **Multivariable model** | **Confessed assaults alone, n =64** | **Witnessed assaults alone, n =82** |
| Age <30 years | 0.7 (0.5–1.0) | - | 0.6 (0.3–1.1) | 0.8 (0.5–1.3) |
| Age ≥30 years | Ref |  | Ref | Ref |
|  |  |  |  |  |
| Males | 0.9 (0.4–1.9) | - | 0.7 (0.3–2.1) | 1.2 (0.4–3.8) |
| Females | Ref |  | Ref | Ref |
|  |  |  |  |  |
| Psychiatric diagnosis | 1.3 (0.7–2.4) | - | 1.5 (0.7–3.4) | 1.4 (0.7–2.9) |
| No psychiatric diagnosis | Ref |  | Ref | Ref |
|  |  |  |  |  |
| Alcohol abuse | 0.7 (0.3–1.5) | - | 0.9 (0.4–2.4) | 0.6 (0.2–1.6) |
| No alcohol abuse | Ref |  | Ref | Ref |
|  |  |  |  |  |
| Narcotic abuse | 1.0 (0.6–1.6) | - | 1.1 (0.5–2.1) | 1.0 (0.5–1.9) |
| No narcotic abuse | Ref |  | Ref | Ref |
|  |  |  |  |  |
| Injury in clothing | 2.3 (0.2–26.6) | 33.5 (0.8–1438.1) | error | error |
| No clothing | error | error | error | error |
| No injury in clothing | Ref | Ref | Ref | Ref |
|  |  |  |  |  |
| Influence of narcotics | 0.6 (0.3–1.2) | 1.8 (1.1–2.9) | 0.4 (0.1–1.3) | 0.5 (0.2–1.3) |
| No influence of narcotics | Ref | Ref | Ref | Ref |
|  |  |  |  |  |
| Penetrating the bones of the ribcage | 1.3 (0.7–2.4) | 1.2 (0.6–2.5) | 1.3 (0.5–3.5) | 1.0 (0.4–2.4) |
| Penetrating the intercostal space | Ref | Ref | Ref | Ref |
|  |  |  |  |  |
| Cranial injury channel | 1.5 (0.6–4.1) | 1.5 (0.5–4.3) | 1.3 (0.3–5.3) | 2.0 (0.6–6.3) |
| Straight horizontal injury channel | error | error | error | error |
| Caudal injury channel | Ref | Ref | Ref | Ref |
|  |  |  |  |  |
| Lateral injury channel | 0.9 (0.2–3.1) | 0.8 (0.2–3.1) | 0.7 (0.1–4.0) | 0.7 (0.1–4.2) |
| Straight sagittal injury channel | error | error | error | error |
| Medial injury channel | Ref | Ref | Ref | Ref |
|  |  |  |  |  |
| Defensive wounds | 0.4 (0.2–1.0) | 0.5 (0.2–1.1) | 0.3 (0.1–1.4) | 0.5 (0.2–1.5) |
| No defensive wounds | Ref | Ref | Ref | Ref |

Univariable logistic regression models presenting non-conclusive associations between variables and corroborated assaults, confessed assaults alone, and witnessed assaults alone using non-corroborated assaults as a reference and a multivariable logistic regression model, adjusting for gender, age, psychiatric diagnosis and and alcohol/narcotic abuse, demonstrating non-conclusive associations between variables and corroborated assaults (ref non-corroborated assaults). Odds ratios (OR) are presented with 95% confidence intervals (CI).

**Article title:** Single Stab Injuries to the Trunk in Survivors of Corroborated Assaults

**Journal name:** International Journal of Legal Medicine

**Author names:** Maria Berg von Linde, MD, Stefan Acosta, MD, PhD, Ardavan M. Khoshnood MD, PhD, Carl Johan Wingren, MD, PhD.

**Affiliation and e-mail address of the corresponding author:** Maria Berg von Linde, MD, Unit for Forensic Medicine, Department of Clinical Sciences Malmö, Faculty of Medicine, Lund University, Sweden. Electronic address: [maria.berg_von_linde@med.lu.se](mailto:maria.berg_von_linde@med.lu.se)
