# Supplementary material for: Single stab injuries to the trunk in survivors of corroborated assaults
Source: Int J Legal Med. 2025 Oct 23;140(2):1121–30. doi: 10.1007/s00414-025-03629-5 (PMC12957011; doi:10.1007/s00414-025-03629-5)
Supplement: Supplementary file 3 — Supplementary Material 3 (DOCX 17.5 KB) [file 414_2025_3629_MOESM3_ESM.docx]

**Table S2. Variables conclusively associated with survived assaults**

|  | Survived assaults, n =385  (ref homicides, n =94), OR (95% CI) | |
| --- | --- | --- |
|  | **Univariable model** | **Multivariable model** |
| Psychiatric diagnosis | 0.05 (0.01–0.1) |  |
| No psychiatric diagnosis | Ref | - |
|  |  |  |
| Alcohol abuse | 0.04 (0.01–0.1) | - |
| No alcohol abuse | Ref |  |
|  |  |  |
| Narcotic abuse | 0.1 (0.04–0.3) | - |
| No narcotic abuse | Ref |  |
|  |  |  |
| Alcohol influence | 0.3 (0.2–0.4) | 0.3 (0.2–0.4) |
| No alcohol influence | Ref | Ref |
|  |  |  |
| Influence of narcotics | 0.1 (0.08–0.2) | 0.1 (0.08–0.2) |
| No influence of narcotics | Ref | Ref |
|  |  |  |
| Abdomen | 1.9 (1.1–3.1) | 1.8 (1.1–3.1) |
| Thorax | Ref | Ref |
|  |  |  |
| The back | 3.7 (1.9–7.2) | 3.8 (1.9–7.5) |
| Right axillary region | 2.1 (0.7–6.1) | 2.2 (0.7–6.8) |
| Left axillary region | 3.7 (1.5–8.8) | 3.8 (1.5–9.1) |
| Frontal trunk | Ref | Ref |
|  |  |  |
| Penetrating the bones of the ribcage | 0.2 (0.1–0.3) | 0.1 (0.1–0.2) |
| Penetrating the intercostal space | Ref | Ref |
|  |  |  |
| Vertical entrance wound | 0.7 (0.4–1.4) | 0.8 (0.4–1.5) |
| Down–right-oriented entrance wound | 0.5 (0.2–0.9) | 0.5 (0.2–0.9) |
| Down–left-oriented entrance wound | 1.1 (0.5–2.3) | 1.2 (0.5–2.6) |
| Horizontal entrance wound | Ref | Ref |

A univariable logistic regression model and a multivariable logistic regression model, adjusting for gender and age, presenting associations between variables, and survived assaults using homicides as a reference. Odds ratios (OR) are presented with 95% confidence intervals (CI).

**Article title:** Single Stab Injuries to the Trunk in Survivors of Corroborated Assaults

**Journal name:** International Journal of Legal Medicine

**Author names:** Maria Berg von Linde, MD, Stefan Acosta, MD, PhD, Ardavan M. Khoshnood MD, PhD, Carl Johan Wingren, MD, PhD.

**Affiliation and e-mail address of the corresponding author:** Maria Berg von Linde, MD, Unit for Forensic Medicine, Department of Clinical Sciences Malmö, Faculty of Medicine, Lund University, Sweden. Electronic address: [maria.berg_von_linde@med.lu.se](mailto:maria.berg_von_linde@med.lu.se)
