# Supplementary material for: Single stab injuries to the trunk in survivors of corroborated assaults
Source: Int J Legal Med. 2025 Oct 23;140(2):1121–30. doi: 10.1007/s00414-025-03629-5 (PMC12957011; doi:10.1007/s00414-025-03629-5)
Supplement: Supplementary file 4 — Supplementary Material 4 (DOCX 16.4 KB) [file 414_2025_3629_MOESM4_ESM.docx]

**Table S3. Variables with no significant differences between survived assaults and homicide**

|  | Survived assaults, n =385 (ref homicides, n =94), OR (95% CI) | |
| --- | --- | --- |
|  | **Univariable model** | **Multivariable model** |
| Age <30 years | 1.1 (0.7–1.7) | - |
| Age ≥30 years | Ref |  |
|  |  |  |
| Males | 1.2 (0.5–2.9) | - |
| Females | Ref |  |
|  |  |  |
| Other indoor location | 0.6 (0.3–1.3) | 0.5 (0.3–1.1) |
| Outdoors | 0.8 (0.4–1.4) | 0.5 (0.3–1.0) |
| Injury inflicted at home | Ref | Ref |
|  |  |  |
| Object found in situ | 0.7 (0.2–2.7) | 0.9 (0.2–3.7) |
| Object found elsewhere | 1.3 (0.8–2.2) | 1.5 (0.9–2.5) |
| No object found | Ref | Ref |
|  |  |  |
| Defensive wounds | 0.7 (0.3–1.4) | 0.7 (0.3–1.5) |
| No defensive wounds | Ref | Ref |

A univariable logistic regression model and a multivariable logistic regression model, adjusting for gender and age, presenting non-conclusive associations between variables and survived assaults using homicides as a reference. Odds ratios (OR) are presented with 95% confidence intervals (CI).

**Article title:** Single Stab Injuries to the Trunk in Survivors of Corroborated Assaults

**Journal name:** International Journal of Legal Medicine

**Author names:** Maria Berg von Linde, MD, Stefan Acosta, MD, PhD, Ardavan M. Khoshnood MD, PhD, Carl Johan Wingren, MD, PhD.

**Affiliation and e-mail address of the corresponding author:** Maria Berg von Linde, MD, Unit for Forensic Medicine, Department of Clinical Sciences Malmö, Faculty of Medicine, Lund University, Sweden. Electronic address: [maria.berg_von_linde@med.lu.se](mailto:maria.berg_von_linde@med.lu.se)
